# Oxidative Stress in Ageing of Hair

**DOI:** 10.4103/0974-7753.51923

**Published:** 2009

**Authors:** Ralph M Trüeb

**Affiliations:** Department of Dermatology, University Hospital of Zurich, Gloriastr. 31, 8091 Zurich, Switzerland

**Keywords:** Androgenetic alopecia, graying, oxidative stress, oral antioxidative supplementation therapy, senescent alopecia, topical melatonin

## Abstract

Experimental evidence supports the hypothesis that oxidative stress plays a major role in the ageing process. Reactive oxygen species are generated by a multitude of endogenous and environmental challenges. Reactive oxygen species or free radicals are highly reactive molecules that can directly damage cellular structural membranes, lipids, proteins, and DNA. The body possesses endogenous defence mechanisms, such as antioxidative enzymes and non-enzymatic antioxidative molecules, protecting it from free radicals by reducing and neutralizing them. With age, the production of free radicals increases, while the endogenous defence mechanisms decrease. This imbalance leads to the progressive damage of cellular structures, presumably resulting in the ageing phenotype. Ageing of hair manifests as decrease of melanocyte function or graying, and decrease in hair production or alopecia. There is circumstantial evidence that oxidative stress may be a pivotal mechanism contributing to hair graying and hair loss. New insights into the role and prevention of oxidative stress could open new strategies for intervention and reversal of the hair graying process and age-dependent alopecia.

## INTRODUCTION

The study of hair focuses on two main streams of interest: On one hand, the esthetic problem of hair and its management, in other words everything that happens outside the skin; on the other hand, the biological problem of hair, in terms of microscopic, biochemical (hormonal, enzymatic), and molecular changes, in other words the 'secret life' of the hair follicle in the depth of the skin. Basic scientists interested in the biology of hair growth and pigmentation have exposed the hair follicle as a highly accessible and unique model that offers unequalled opportunities also to the gerontologist for the study of environmental and age-related effects. Its complex multicell type interaction system involving epithelium, mesenchyme, and neuroectoderm, and its unique cyclical activity of growth, regression, rest, and regrowth provides the investigator with a range of stem, differentiating, mitotic and postmitotic terminally differentiated cells, including cells with variable susceptibility to apoptosis, for study. Finally, a number of intrinsic and extrinsic modulating factors for hair growth and pigmentation have been identified and are being further tested *in vitro*.[1]

## AGEING OF HAIR

Ageing is a complex process involving various genetic, hormonal, and environmental mechanisms. As the rest of the skin, the scalp and hair are subject to intrinsic or chronologic ageing, and extrinsic ageing due to environmental factors. Both occur in conjunction with the other and are superimposed on each other. Intrinsic factors are related to individual genetic and epigenetic mechanisms with interindividual variation. Examples of intrinsic factors are familial premature graying and androgenetic alopecia (AGA). Extrinsic factors include ultraviolet radiation (UVR), smoking, and nutrition.

Experimental evidence supports the hypothesis that oxidative stress plays a major role in the ageing process. As early as 1956, Harman *et al*.[2] first proposed this 'free radical theory of aging'. Today it is one of the most widely accepted theories used to explain mechanisms underlying the ageing process. Free radicals are highly reactive molecules with unpaired electrons that can directly damage various cellular structural membranes, lipids, proteins, and DNA. The damaging effects of these reactive oxygen species are induced internally during normal metabolism and externally through exposure to various oxidative stresses from the environment. The body possesses endogenous defence mechanisms, such as antioxidative enzymes (superoxide dismutase, catalase, glutathione peroxidase) and non-enzymatic antioxidative molecules (vitamin E, vitamin C, glutathione, ubiquinone), protecting it from free radicals by reducing and neutralizing them.[3] With age, the production of free radicals increases, while the endogenous defence mechanisms decrease. This imbalance leads to the progressive damage of cellular structures, presumably resulting in the ageing phenotype. The ageing phenotype of hair manifests as decrease of melanocyte function or graying, and decrease in hair production or alopecia.

## GRAYING

Hair graying (canities) is a natural age-associated feature. The hair graying trait correlates closely with chronological ageing, but it occurs to varying degrees in all individuals. Hair is said to gray prematurely if it occurs before the age of 20 in Caucasians and before 30 in Africans. While premature canities more commonly appear without underlying pathology, presumably inherited in an autosomal dominant manner, it has been linked to a similar cluster of autoimmune disorders observed in association with vitiligo, i.e., pernicious anemia and autoimmune thyroid disease, and several rare premature ageing syndromes, such as Werner's syndrome. Although graying is understood as a loss of pigment in the shaft, its cellular and molecular origins are incompletely understood.[1] Theories for the gradual loss of pigmentation include exhaustion of enzymes involved in melanogenesis, impaired DNA repair, loss of telomerase, antioxidant mechanisms, and antiapoptotic signals.

The colour of hair mainly relies on the presence or absence of melanin pigment. Skin and hair melanins are formed in cytoplasmic organelles called melanosomes, produced by the melanocytes, and are the product of a complex biochemical pathway (melanogenesis) with tyrosinase being the rate-limiting enzyme. So far, the process of hair graying has been attributed to the loss of the pigment-forming melanocytes from the ageing hair follicle.[4] The net effect of this reduction is that fewer melanosomes are incorporated into cortical keratinocytes of the hair shaft. In addition, there also appears to be a defect of melanosome transfer, as keratinocytes may not contain melanin despite their proximity to melanocytes with remaining melanosomes. This defect is further corroborated by the observation of melanin debris in and sometimes around the graying hair bulb. This anomaly is due to either defective melanosomal transfer to the cortical keratinocytes or melanin incontinence due to melanocyte degeneration. Eventually, no melanogenic melanocytes remain in the hair bulb. This decrease of melanin synthesis is associated with a decrease in tyrosinase activity. Ultrastructural studies have shown that remaining melanocytes not only contain fewer melanosomes, but the residual melanosomes may be packaged within autophagolysosomes. This removal of melanosomes into autophagolysosomes suggests that they are defective, possibly with reactive melanin metabolites. This interpretation is supported by the observation that melanocytes in graying hair bulbs are frequently highly vacuolated, a common cellular response to increased oxidative stress. By analogy with the free radical theory of ageing, a 'free radical theory of graying' has been proposed. [5] The extraordinary melanogenic activity of pigmented bulbar melanocytes, continuing for up to 10 years in some hair follicles, is likely to generate large amounts of reactive oxygen species via the hydroxylation of tyrosine and the oxidation of DOPA to melanin. If not adequately removed by an efficient antioxidant system, an accumulation of these reactive oxidative species will generate significant oxidative stress. It is possible that the antioxidant system becomes impaired with age leading to damage to the melanocyte itself from its own melanogenesis-related oxidative stress. Since mutations occur at a higher rate in tissue exposed to high levels of oxidative stress, and these accumulate with age, the induction of replicative senescence with apoptosis is likely to be an important protective mechanism against cell transformation.

Wood *et al*.[6] have recently demonstrated for the first time that human white scalp hair shafts accumulate hydrogen peroxide (H_2_O_2_) in millimolar concentrations, and almost absent catalase and methionine sulfoxide reductase (MSR) protein expression in association with functional loss of methionine sulfoxide repair in the entire gray hair follicle. Accordingly, methionine sulfoxide formation of methionine residues (Met), including Met 374 in the active site of tyrosinase, the key enzyme in melanogenesis, limits enzyme functionality, which eventually leads to loss of hair color. While the entire hair follicles are subject to H_2_O_2_-mediated stress, it is tempting to assume that, besides tyrosinase and MSR, other proteins and peptides, including antiapoptotic Bcl-2 protein, are targets for oxidation, which in turn could explain melanocyte apoptosis in the gray hair follicle. Moreover, H_2_O_2_-mediated oxidation has been documented for many other important regulators of pigmentation, including the proopiomelanocortins α-melanocyte-stimulating hormone and β-endorphin,[7] the prohormone convertases,[8] and the synthesis and recycling of the ubiquitous cofactor 6-tetrahydrobiopterin.[9] Although as yet little published data is available on the hair follicle melanocyte stem cell population, it is tempting to speculate that these cells may well also be target to oxidation. Since the discovery of unpigmented melanocyte stem cells located within the hair follicle,[10] the question has arisen whether the process underlying hair graying arises specifically from changes in differentiated, pigmented melanocytes or the unpigmented progenitors which provides them. Utilizing melanocyte-tagged transgenic mice and ageing human hair follicles, Nishimura *et al*.[11] have recently demonstrated that hair graying may be caused by defective self-maintenance of melanocyte stem cells, and not of differentiated melanocytes. This process was dramatically accelerated with Bcl-2-deficiency, which causes selective apoptosis of melanocyte stem cells.

Finally, anecdotal evidence indicates that gray hair is coarser and less manageable than pigmented hair. Gray hair is said to often fail to hold a temporary or permanent set, and to be more resistant to incorporating artificial color, both of which suggest changes to the underlying substructure of the hair fiber. Gray hair has been found to have increased sensitivity to weathering, increased cysteic acid residues and decreased cystine, and increased fiber reactivity to reducing and oxidizing agents.[12] Given the close interaction of melanin transferring melanocytes with hair shaft-forming precortical keratinocytes, it is conceivable that other functions of these cell types are affected by this activity. One possibility is that melanin transfer decreases keratinocyte turnover and increases keratinocyte terminal differentiation. Indeed, white beard hair has been shown to grow up to four times the rate of adjacent pigmented hair.[13] In this way, aging hair follicles may reprogram their matrix keratinocytes to increase production of medullary, rather than cortical, keratinocytes. In fact the medulla is often enlarged and collapsed, forming a central cavity in gray and white hairs.[14,15] An evolutionary basis for this increased medullation in senile white hair may reflect the enhanced insulation provided by these hairs which would confer an important benefit for temperature regulation. In this way, it may compensate for the loss the sunlight-absorbing and thus heat-trapping properties of melanized dark hair.[1] Whether these differences, seemingly related to the lack of melanin and to the enlarged medulla, are also directly responsible for the coarseness of white hair and their relative resistance to hair setting and coloring is not clearly established.

## ANDROGENETIC ALOPECIA

The AGA is a heritable, androgen- and age-dependent process resulting in progressive decline in visible scalp hair density in a sex-dependent defined pattern. It affects at least 50% of men by the age of 50 years, and up to 70% of all males in later life.[16] Estimates of its prevalence in women have varied widely, though recent studies claim that 16% of women aged under 50 years are affected, increasing to a proportion of 30–40% of women aged 70 years and over.[17] It is assumed that the genetically predisposed hair follicles are the target for androgen-stimulated hair follicle miniaturization, leading to gradual replacement of large, pigmented hairs (terminal hairs) by barely visible, depigmented hairs (vellus hairs) in affected areas.[18] Affected men typically develop bitemporal recession of hair and vertex balding (male pattern AGA), while women present with diffuse thinning of the crown and an intact frontal hair line (female pattern AGA). Though AGA may manifest as early as at the age of 16 years (before 16 years, it is called by definition premature alopecia), balding has traditionally been considered as an attribute of ageing at all times and in all cultures. Indeed, evidence is emerging that AGA may be considered a form of organ-specific premature ageing.

While the genetic involvement in AGA is pronounced, it remains poorly understood. Major advances have been achieved in our understanding of peculiarities of the androgen metabolism involved in the pathogenesis of AGA[19]: Androgen-dependent processes are predominantly due to the binding of dihydrotestosterone (DHT) to the androgen receptor (AR). DHT-dependent cell functions depend on the availability of weak androgens, their conversion to more potent androgens via the action of 5a-reductase, low enzymatic activity of androgen inactivating enzymes, and functionally active AR present in high numbers. The predisposed scalp exhibits high levels of DHT, and increased expression of the AR. Conversion of testosterone to DHT within the dermal papilla plays a central role, while androgen-regulated factors deriving from dermal papilla cells are believed to influence growth of other components of the hair follicle. Since many extrinsic hair growth-modulatory factors, such as androgens,[20] apparently operate at least in part via the dermal papilla, research is currently also focused on identifying androgen-regulated factors deriving from dermal papilla cells. Of the several factors that have been suggested to play a role in hair growth, so far only insulin-like growth factor (IGF-1) has been reported as altered *in vitro* by androgens,[21] and stem cell factor (SCF) has been found to be produced in higher amounts by androgen-dependent beard cells than in control nonbalding scalp cells, presumably also in response to androgens.[22] Since SCF is the ligand for the cell surface receptor c-kit on melanocytes, this may also play a role for hair pigmentation.

Nevertheless, the limited success rate of treatment of AGA with hair growth promoters, such as topical minoxidil, or modulators of androgen metabolism, such as finasteride, means that further pathogenic pathways must be taken into account.

Recent studies have focused on oxidative stress: Naito *et al*.[23] have recently analyzed the effect of the lipid peroxides on hair follicles, and observed that the topical application of linolein hydroperoxides, one of the lipid peroxides, lead to the early onset of the catagen phase in murine hair cycles. Furthermore, they found that lipid peroxides induced apoptosis of hair follicle cells. They also induced apoptosis in human epidermal keratinocytes by up-regulating apoptosis-related genes. These results indicate that lipid peroxides, which can cause free radicals, induce the apoptosis of hair follicle cells, and this is followed by early onset of the catagen phase. Bahta *et al*.[24] cultured dermal hair papilla cells (DPC) from balding and nonbalding scalp and demonstrated that balding DPCs grow slower *in vitro* than nonbalding DPCs. Loss of proliferative capacity of balding DPCs was associated with changes in cell morphology, expression of senescence-associated beta-galactosidase, decreased expression of proliferating cell nuclear antigen and Bmi-1, upregulation of p16 (INK4a)/pRb and nuclear expression of markers of oxidative stress and DNA damage including heat shock protein-27, super oxide dismutase catalase, ataxia-telangiectasia-mutated kinase (ATM), and ATM- and Rad3-related protein. The finding of premature senescence of balding *DPC in vitro* in association with expression of p16(INK4a)/pRB suggests that balding DPCs are particularly sensitive to environmental stress.

## INFLAMMATORY PHENOMENA AND FIBROSIS

The implication of microscopic follicular inflammation in the pathogenesis of AGA has emerged from several independent studies: An early study referred to an inflammatory infiltrate of activated T cells and macrophages in the upper third of the hair follicles, associated with an enlargement of the follicular dermal sheath composed of collagen bundles (perifollicular fibrosis), in regions of actively progressing alopecia.[25] Horizontal section studies of scalp biopsies indicated that the perifollicular fibrosis is generally mild, consisting of loose, concentric layers of collagen that must be distinguished from cicatricial alopecia.[26] The term 'microinflammation' has been proposed, because the process involves a slow, subtle, and indolent course, in contrast to the inflammatory and destructive process in the classical inflammatory scarring alopecias.[27] The significance of these findings has remained controversial. However, morphometric studies in patients with male pattern AGA treated with minoxidil showed that 55% of those with microinflammation had regrowth in response to treatment, in comparison to 77% in those patients without inflammation and fibrosis.[26] Moreover, some forms of primary fibrosing alopecia may represent pathological exaggeration of AGA associated with follicular inflammation and fibrosis, specifically postmenopausal frontal fibrosing alopecia,[28] and fibrosing alopecia in a pattern distribution.[29]

An important question is how the inflammatory reaction pattern is generated around the individual hair follicle. Inflammation is regarded as a multistep process which may start from a primary event. Some authors proposed that alopecia may result from cumulative physiological degeneration of selected hair follicles. They described in healthy murine skin clusters of perifollicular macrophages as perhaps indicating the existence of a physiological program of immunologically controlled hair follicle degeneration by which malfunctioning follicles are removed by programmed organ deletion, and suggested that perhaps an exaggerated form of this process might underlie some forms of primary scarring alopecia.[30] The observation of a perifollicular infiltrate in the upper follicle near the infundibulum of human hair follicles in AGA suggests that the primary causal event for the triggering of inflammation might occur near the infundibulum.[27] On the basis of this localization and the microbial colonization of the follicular infundibulum with *Propionibacterium* sp., *Staphylococcus* sp., *Malassezia* sp., or other members of the transient flora, one could speculate that microbial toxins or antigens could be involved in the generation of the inflammatory response. Alternatively, keratinocytes themselves may respond to oxidative stress from irritants, pollutants, and UV irradiation, by producing nitric oxide, and by releasing intracellularly stored IL-1α. This pro-inflammatory cytokine by itself has been shown to inhibit the growth of isolated hair follicles in culture. [31] Moreover, adjacent keratinocytes, which express receptors for IL-1, start to engage the transcription of IL-1 responsive genes: mRNA coding for IL-1β, TNFα, and IL-1α, and for specific chemokine genes, such as IL-8, and monocyte chemoattractant protein-1 (MCP-1) and MCP-3, themselves mediators for the recruitment of neutrophils and macrophages, have been shown to be upregulated in the epithelial compartment of the human hair follicle.[27] Besides, adjacent fibroblasts are also fully equipped to respond to such a pro-inflammatory signal. The upregulation of adhesion molecules for blood-borne cells in the capillary endothelia, together with the chemokine gradient, drives the transendothelial migration of inflammatory cells, which include neutrophils through the action of IL-8, T cells, and Langerhans cells at least in part through the action of MCP-1. After processing of localized antigen, Langerhans cells, or alternatively keratinocytes, which may also have antigen presenting capabilities, could then present antigen to newly infiltrating T lymphocytes and induce T-cell proliferation. The antigens are selectively destroyed by infiltrating macrophages, or natural killer cells. On the occasion that the causal agents persist, sustained inflammation is the result, together with connective tissue remodeling, where collagenases, such as matrix metalloproteinase (also transcriptionally driven by pro-inflammatory cytokines) play an active role.[27] Collagenases are suspected to contribute to the tissue changes in perifollicular fibrosis.

## SENESCENT ALOPECIA

Senescent alopecia has been defined as nonandrogen- dependent hair thinning found in those over 60 years of age. Much like AGA, it involves a progressive decrease in the number of anagen follicles and hair diameter.[32] It frequently occurs together with AGA, further complicating its delineation from the latter, though recent data comparing AGA and senescent alopecia using microarray analysis have demonstrated significant differences in the gene expression patterns suggesting they represent different entities: In AGA, genes required for anagen onset (Wnt-beta-catenin, TGF-alpha, TGF-beta, Stat-3, Stat-1), epithelial signal to dermal papilla (PPARd, IGF-1), hair shaft differentiation (Notch, Msx2, KRTs, KAPs), and anagen maintenance (Msx2, Activin, IGF-1) were downregulated, while genes for catagen (BDNF, BMP2, BMP7, VDR, IL-1, ER) and telogen induction and maintenance (VDR, RAR) were upregulated. In senescent alopecia, genes involved in epithelial signal to dermal papilla (FGF5), actin cytoskeleton (DST, ACTN2, TNNI3, PARVB), and mitochondrial function (JAK2, PRKD3, AK2, TRAP1, TRIO, ATP12A, MLL4, STK22B) were downregulated, while oxidative stress and inflammatory response genes were upregulated.[33]

In their study on aging and hair cycles over an exceptionally long duration of 8–14 years, Courtois *et al*.[34] found a reduction in the duration of hair growth and in the diameter of hair shafts, and a prolongation of the interval separating the loss of a hair in telogen and the emergence of a replacement hair in anagen (latency phase). These phenomena resemble those observed in the course of AGA, although their development is less marked, suggesting AGA a premature aging phenomenon. This aging process was evidenced by a reduction in the number of hairs per unit area and deterioration in the quality of scalp hair. The reduction in density was manifested to different degrees in different individuals. It amounted to less than 10% in 10 years in the individuals with the least alopecia, and was much more pronounced in the balding subjects. The maximal length of hair diminished as the subjects aged, in parallel the hairs became finer. However, among nonbalding subjects, there was a tendency for the proportion of thicker hairs to increase. Finally, aging did not appear to follow a perfectly regular course over time. Periods of stability, or even partial remission, alternated with periods of more marked evolution, reflecting perhaps the influence of individual factors such as the subject's general health, and risk factors for accelerated aging. In a more recent study on scalp hair follicle density in four-millimeter punch biopsies obtained from a cohort of 928 different women aged between 13 and 84 years visiting their hair consultation unit for evaluation of hair loss, Sinclair *et al*.[35] found for every one-year increase in age a decrease in the total number of hair follicles of 0.093. Over 10 years, a patient from their cohort was expected to lose 0.76 hair follicles per four-millimeter punch biopsy, and over 53 years four hair follicles per biopsy. They interpreted the order of magnitude of the decline in hair follicle numbers to be similar to the 15% reduction in eccrine gland number, the 10–20% reduction per decade of enzymatically active melanocytes, the 20–50% reduction in morphologically identifiable Langerhans cells, and the 20% loss of dermal thickness in the elderly,[36] and concluded that in the absence of AGA, visible hair loss is likely to be limited.

## EFFECTS OF SMOKING AND UVR

Besides being the single most preventable cause of significant morbidity and an important cause of death in the general population, tobacco smoking has been associated with adverse effects on the skin. While smoke-induced premature skin ageing has long attracted the attention of the medical community, Mosley and Gibbs[37] were the first to indicate a relationship between smoking and graying of hair and alopecia in men. The number of studied women was not sufficient enough to draw any significant conclusion. An association of smoking status with AGA was recently confirmed in an Asian community[38]: Smoking status, current amount of cigarette smoking, and smoking intensity were statistically significant factors responsible for AGA in men after controlling for age and family history. So far, no data exist for women or partners of heavy smokers via secondary inhalation. The mechanisms by which smoking causes hair loss are multifactorial, and probably related to effects of cigarette smoke on the microvasculature of the dermal hair papilla, smoke genotoxicants causing damage to DNA of the hair follicle, smoke-induced imbalance in the follicular protease/ antiprotease systems controlling tissue remodeling during the hair growth cycle, pro-oxidant effects of smoking leading to the release of pro-inflammatory cytokines resulting in follicular microinflammation and perifollicular fibrosis, and finally increased hydroxylation of estradiol creating a relative hypoestrogenic state (overview[39]). The fact that cigarette smoke-associated hair loss is of the androgenetic type again indicates that genetic factors contribute. The recent findings of Bahta *et al*.[24] point to the fact that DPCs of androgenetic hair follicles are more sensitive to environmental oxidative stress.

Progressive thinning of scalp hair with age results in a gradual decline in natural protection of the scalp from UVR. While it is has been recognized that at least 50% of UVR-induced damage to the skin is attributable to the UVR-induced formation of free radicals,[40] the effects of UVR on hair have received less attention. Photochemical impairment of the hair includes degradation and loss of hair proteins as well as degradation of hair pigment. UVB radiation is responsible for hair protein loss and UVA radiation is responsible for hair colour changes.[41‐43] Absorption of radiation in photosensitive amino acids of the hair and their photochemical degradation is producing free radicals. They have adverse impact on hair proteins, especially keratin. Melanin can partially immobilize free radicals and block their entrance in keratin matrix. Moreover, clinical observations and theoretical considerations suggest that UVR may also have negative effects on hair growth (overview [44]): Acute telogen effluvium from UVR has been described,[45] and the production of porphyrins by *Propionibacterium* sp. in the pilosebaceous duct, with photoactivation of porphyrins[46] leading to oxidative tissue injury, may contribute to follicular microinflammation operative at the level of the follicular stem cells. Histopathologically elastosis is regularly found in scalp biopsies, especially in alopecic conditions. A recent study demonstrated a relationship between the degree of scalp elastosis and severity of AGA:[47] The scalp dermis was significantly thicker in AGA than in unaffected control subjects. The difference was due to severer elastosis in baldness. The earliest signs of solar elastosis preceded hair thinning. When elastosis was thicker than 0.2 mm, a negative exponential correlation was found between hair diameter and severity of solar elastosis. So far, the incidence of AGA in relation to the level of UVR exposure in different parts of the world has not been studied, but may be confounded by ethnic factors.

## PHOTOPROTECTION

As a consequence of increased leisure time with a growing popularity of outdoor activities and holidays in the sun, awareness of sun protection has become imperative. Topically applied chemicals that act as sun protectors are widely utilized and offer the most convenient means of protecting the glabrous skin against acute (sunburn) and chronic pathologic effects of UVR. Out of cosmetic reasons their use on the hair-bearing scalp is problematic, unless complete baldness is present. Although hats provide the best protection of the scalp from UVR, not all patients find it convenient or acceptable for this purpose. While protection of the hair against photodamage has been extensively studied, there are no data on photoprotection of the hair-bearing scalp: It has been found that hair dyes may protect hair against photodamage;[48] recent experimental work indicates that cinnamidpropyltrimonium chloride, a quaternized UV absorber, delivered from a shampoo system, is suitable for photoprotection of hair, while simultaneously providing an additional conditional benefit on hair;[49] and solid lipid nanoparticles have been developed as novel carriers of UV blockers for the use on skin and hair, while offering photoprotection on their own by reflecting and scattering UVR.[50]

## POTENTIAL ROLE OF ORAL SUPPLEMENTATION THERAPY

Systemic photoprotection has been the focus of more recent investigation, in as much as this would overcome some of the problems associated with the topical use of sunscreens. Since the antioxidant defence mechanisms decrease as part of the natural ageing process, and are inhibited by UVR,[51] while the production of reactive oxygen species increases, it seems reasonable to substitute antioxidants. Preclinical studies illustrate some photoprotective properties of supplemented antioxidants,[52‐54] though the effect is weak. Moreover, there is a paucity of controlled clinical trials in humans examining the role of antioxidants in preventing or decelerating skin ageing. Therefore, further experimental data need to be generated. Current research suggests that combinations of different antioxidants may have synergistic effects and better efficacy, when compared with single antioxidants used for photoprotection.[55,56]

Since concentration-dependent H_2_O_2_-mediated oxidation of tyrosinase in hair follicle melanocytes, in association with the loss of functioning methionine sulfoxide repair, sheds a new light on the slowing down of hair pigmentation as observed in the age-dependent graying process, and under *in vitro* condition, methionine oxidation can be prevented by l-methionine, it would be interesting whether l-methionine could be useful for intervention or reversal of the hair graying process.[6]

With respect cigarette smoke-induced alopecia, d'Agostini *et al*.[57] demonstrated that high doses of environmental cigarette smoke induce alopecia in mice. This effect was prevented by the oral administration of a mixture of l-cystine with vitamin B6.[58] Combinations of l-cystine and B vitamins are traditionally used OTC products for the treatment of hair loss. Their effect on humans exposed to cigarette smoke and losing hair has so far not been studied. Oral supplementation with l-cystine, pantothenic acid, thiamine nitrate, and medicinal yeast has been shown to increase the anagen rate in apparently healthy women with telogen effluvium in a placebo-controlled study.[59]

## POTENTIAL ROLE OF TOPICAL MELATONIN

Finally, among other natural substances, topical melatonin seems to be a promising candidate. Melatonin acts as a potent antioxidant,[60,61] direct radical scavenger,[62] and antiaging factor.[63,64] In the skin, melatonin is present in a melatoninergic system that is fully expressed in humans.[65] Biological effects of melatonin on cell growth regulation have been shown in human keratinocytes.[66,67] Furthermore, in healthy human subjects, topically melatonin effectively prevented the development of UV-induced erythema. [68,69] Similarly, cell death of UVR irradiated leukocytes was prevented by melatonin through the scavenging of reactive oxygen species.[70‐72] In the latter study, the antioxidative effects of melatonin were superior to those exerted by vitamin C. Thus, the melatoninergic system in the skin may counteract the effects of environmental stressors to preserve the functional integrity and maintain the homeostasis of the skin and hair. In contrast to topical minoxidil and oral finasteride in the management of AGA, topical melatonin would seem to represent the first topical 'antiaging' product for treatment of the ageing scalp. Penetration and bioavailability studies (unpublished data) have so far been done in the forefront of a pilot study by Fischer *et al*.[73] suggesting that topically applied melatonin might influence human hair growth *in vivo*.

## CONCLUSION

The condition of the hair has been at the center of attention of human civilization since ancient times. Hair ageing comprises decrease in hair pigmentation (graying) and decrease in hair production (alopecia). Like the skin, the hair follicle is subject to intrinsic and extrinsic ageing. Intrinsic factors are related to individual genetic and epigenetic mechanisms with interindividual variation. Examples are familial premature graying and AGA. Extrinsic factors include smoking and UVR. Experimental evidence supports the hypothesis that oxidative stress also plays a role in the ageing process of the hair follicle. Reactive oxygen species are generated by a multitude of endogenous and environmental challenges. The body possesses endogenous defence mechanisms, such as antioxidative enzymes and non-enzymatic antioxidative molecules, protecting it from free radicals by reducing and neutralizing them. With age, the production of free radicals increases, while the endogenous defence mechanisms decrease. This imbalance leads to the progressive damage of cellular and molecular structures, presumably resulting in the ageing phenotype. New insights into the role and prevention of oxidative stress could open new strategies for intervention and reversal of the hair graying process and age-dependent alopecia. Topical antiaging compounds that are currently under investigation include photoprotectors, such as cinnamidpropyltrimonium chloride and solid lipid nanoparticles as carriers for UV blockers, oral supplementation with l-cystine and l-methionine, and topical melatonin.
